# Effects of PCNL under the guidance of hologram technology on stress response and renal injury factors in patients with complex kidney stones

**DOI:** 10.5937/jomb0-48327

**Published:** 2024-06-15

**Authors:** Changming Liu, Zimin Dong, Mingxiong Sheng, Xinghua Huang, Youfeng Huang

**Affiliations:** 1 Mindong Hospital Affiliated to Fujian Medical University, Department of Urology, Fujian, China

**Keywords:** complex kidney stones, holographic image technology, percutaneous nephrolithotripsy, stress response, kidney injury factor, kidney function, inflammatory response, complication, složeni kamen u bubregu, tehnologija holografskih slika, perkutana nefrolitotripsija, odgovor na stres, faktor povrede bubrega, funkcija bubrega, zapaljenski odgovor, komplikacija

## Abstract

**Background:**

To investigate the effect of percutaneous nephrolithotomy (PCNL) guided by holographic image technology on stress response and renal injury factors in patients with complex renal calculi.

**Methods:**

A retrospective analysis was conducted on the clinical data of 70 patients admitted to our hospital between August 2022 and June 2023 who had complex kidney stones. The patients were divided into two groups, namely, group A and group B, based on whether they received guidance from preoperative holographic imaging technology. Group A consisted of forty patients who underwent PCNL after undergoing renal CT examination prior to surgery, while Group B included thirty patients who underwent PCNL guided by holographic imaging technology. Various indexes, including operative factors, stress response, inflammatory response, renal injury factors, renal function, complication rate, and the rate of achieving complete stone clearance in a single procedure, were compared between the two groups.

**Results:**

In group B, the puncture time and operation time of the target calyces were shorter compared to group A, additionally, the intraoperative blood loss in group B was lower than that in group A (P<0.05). 24 h after surgery, group B exhibited higher levels of superoxide dismutase (SOD) and glutathione peroxidase (GSH-Px) compared to group A, additionally, the level of malondialdehyde (MDA) in group B was lower than that in group A (P<0.05). 24 h after surgery, group B exhibited lower levels of tumor necrosis factor a (TNF-a), interleukin-6 (IL-6), IL-1, and hypersensitive C-reactive protein (hs-CRP) compared to group A (P<0.05). Furthermore, group B showed lower levels of neutrophil gelatinase-associated lipid carrier protein (NGAL), inducible protein-8-like molecule 2 (TIPE2), and b2-microglobulin (b2-MG) than group A at the 7-day mark (P<0.05). 24 h after the operation, There was no statistically significant difference observed in the levels of SCr, BUN, between group A and group B group (P > 0.05); however, exhibited lower levels of CysC compared to group A (P < 0.05). Additionally, there were no significant differences in postoperative complications between group B and group A (P>0.05). Furthermore, one month after surgery, the one-time stone clearance rate in group B was significantly higher than that in group A (P<0.05).

**Conclusions:**

PCNL under the guidance of hologram technology can shorten the time of puncture target calyce and operation, reduce the amount of intraoperative blood loss, effectively reduce the postoperative stress reaction and inflammatory reaction of patients, reduce the level of renal injury factors, improve renal function, and increase the one-time stone clearance rate.

## Abstract (sr)

**Uvod:**

Cilj je bio da se istraži efekat perkutane nefrolitotomije (PCNL) vođene tehnologijom holografske slike kao odgovor na stres i faktore oštećenja bubrega kod pacijenata sa složenim bubrežnim kamencima.

**Metode:**

Urađena je retrospektivna analiza kliničkih podataka 70 pacijenata koji su primljeni u našu bolnicu u periodu od avgusta 2022. do juna 2023. godine sa složenim kamenom u bubregu. Pacijenti su podeljeni u dve grupe, i to u grupu A i grupu B, na osnovu toga da li su dobili uputstva od preoperativne tehnologije holografskog snimanja. Grupu A činilo je četrdeset pacijenata koji su podvrgnuti PCNL-u nakon renalnog CT pregleda pre operacije, dok je Grupa B obuhvatala trideset pacijenata koji su podvrgnuti PCNL-u vođenom tehnologijom holografskog snimanja. Različiti indeksi, uključujući operativne faktore, odgovor na stres, inflamatorni odgovor, faktore oštećenja bubrega, funkciju bubrega, stopu komplikacija i stopu postizanja potpunog uklanjanja kamenca u jednoj proceduri, upoređivani su između dve grupe.

**Rezultati:**

U grupi B vreme punkcije i vreme operacije ciljnih čašica je bilo kraće u odnosu na grupu A, pri čemu je intraoperativni gubitak krvi u grupi B bio manji nego u grupi A (P<0,05). 24 h nakon operacije, grupa B je pokazala više nivoe superoksid dismutaze (SOD) i glutation peroksidaze (GSH-Pk) u poređenju sa grupom A, dodatno, nivo malondialdehida (MDA) u grupi B je bio niži nego u grupi A (P <0,05). 24 h nakon operacije, grupa B je pokazala niže nivoe faktora tumorske nekroze a (TNF-a), interleukina-6 (IL-6), IL-1 i preosetljivog C-reaktivnog proteina (hs-CRP) u poređenju sa grupom A (P<0,05). Pored toga, grupa B je pokazala niže nivoe proteina nosača lipida (NGAL) povezanog sa neutrofilom želatinazom, inducibilnog molekula 2 sličnog proteinu-8 (TIPE2) i b2-mikroglobulina (b2-MG) od grupe A na 7-dnevnom nivou ( P<0,05). 24 h nakon operacije, nije uočena statistički značajna razlika u nivoima SCr, BUN između grupe A i grupe B (P > 0,05); međutim, pokazali su niže nivoe CisC u poređenju sa grupom A (P <0,05). Pored toga, nije bilo značajnih razlika u postoperativnim komplikacijama između grupe B i grupe A (P>0,05). taviše, mesec dana nakon operacije, jednokratna stopa uklanjanja kamenca u grupi B bila je značajno veća nego u grupi A (P<0,05).

**Zaključak:**

PCNL pod vodstvom hologramske tehnologije može skratiti vrijeme punkcije ciljne čašice i operacije, smanjiti količinu intraoperativnog gubitka krvi, efikasno smanjiti postoperativnu stresnu reakciju i inflamatornu reakciju pacijenata, smanjiti nivo faktora oštećenja bubrega, poboljšati rad bubrega, funkciju i povećati jednokratnu stopu čišćenja kamena.

## Introduction

Complex renal calculi are a prevalent condition in urology, and their incidence has been steadily increasing in recent years. If not treated appropriately, it can result in urinary tract infection, urinary tract obstruction, and even severe renal parenchyma atrophy, leading to significant impairment of renal function [1] [2]. Currently, minimally invasive percutaneous nephrolithotomy (PCNL) has made significant advancements and is now the primary treatment for complex renal calculi. Research has indicated that the surgical outcomes, postoperative complications, and stone clearance rate following PCNL are closely dependent on the selection and establishment of renal puncture paths [3]. At present, the international standard method for intraoperative calyceal puncture in patients with complex kidney stones is to combine the preliminary assessment of preoperative renal CT examination with the use of ultrasound, fluoroscopy, or a combination of both during the operation to guide the puncture [4]. However, both ultrasound and fluoroscopy only present two-dimensional planes, and sometimes it is difficult for clinicians to obtain satisfactory results in terms of clarity and resolution. The assessment of stone conditions largely depends on the doctors’ personal understanding of anatomy and processing of image information, with large subjective factors [5]. Inappropriate intraoperative traumatic puncture and other surgical procedures may cause severe oxidative stress response, inflammatory response and kidney injury after surgery, which is not conducive to the prognosis of patients. Therefore, how to carry out preoperative planning and intraoperative guidance for complex renal calculi to ensure the surgical effect is worthy of clinical research. With the continuous development of artificial intelligence technology, holographic imaging technology is increasingly widely used in medicine. Holographic image is a 3D model of surface drawing with powerful interactive functions, after boundary recognition and other segmentation processing, the two-dimensional image is restored, and the target organ is restored in a surface manner, which is gradually used in the practice of renal surgery [6]. Building upon this foundation, the present study conducted a retrospective analysis of clinical data from 70 patients diagnosed with complex kidney stones. The objective was to evaluate the safety and efficacy of percutaneous nephrolithotomy (PCNL) surgery in the management of complex kidney stones utilizing holographic imaging technology as a guiding tool. The findings are presented below.

## Materials and methods

### General information

A retrospective analysis was performed on the clinical data of 70 patients who were admitted to our hospital with complex kidney stones during the period from August 2022 and June 2023. These patients were classified into two groups, namely group A and group B, based on whether they received preoperative guidance using holographic image technology. The general data of both groups were carefully balanced (*P* > 0.05) and are presented in Table 1 for comparative analysis.

**Table 1:** Patient and stones data.

Baseline Data		group A (n=40)	group B (n=30)	Value of statistics	P
Age (x̄ ±S, years of age)		45.24±3.26	45.08±3.41	t=0.199	0.843
Course of disease (x̄ ±S, years)		3.26±0.52	3.24±0.59	t=0.0150	0.881
Gender (example (%))	male	25 (62.50)	18 (60.00)	χ^2^=0.045	0.832
	female	15 (37.50)	12 (40.00)		
Affected side (case (%))	Left side	20 (50.00)	16 (53.33)	χ^2^=0.076	0.782
	Right side	20 (50.00)	14(46.67)		
Body mass index (x̄ ±S, kg/m^2^)		23.69±1.52	23.66±1.53	t=0.082	0.935
Maximum stone diameter (x̄ ±S, mm)		26.11±3.54	26.14±3.56	t=0.035	0.972
Stone type (case (%))	Staghorn renal calculi	15 (37.50)	12 (40.00)	χ^2^=0.246	0.970
	Huge cast kidney stone	12 (30.00)	8 (26.67)		
	Renal calculi in solitary	11 (27.50)	9 (30.00)		
	Horseshoe kidney kidney	2 (5.00)	1 (3.33)		

### Inclusion criteria

(1) Inclusion criteria: The diagnosis of renal complex renal calculi was confirmed by imaging examination. Additionally, complete clinical data were available for all patients. Only individuals with normal cognitive function, communication ability, visual acuity, and hearing were considered for inclusion. The age range of the participants was between 30 and 70 years.

(2) Exclusion criteria: Patients were excluded from the study if they had concomitant malignant tumors, hematological diseases, or severe heart, liver, lung, or kidney dysfunction. Pregnant and lactating women were also excluded. Patients with abnormal coagulation function or a tendency to bleed, secondary glomerular disease, uncontrolled infection or infectious diseases, or urinary tract infection were not included in the analysis.

### Methods

Group A underwent a preoperative routine renal CT examination to determine the condition of calculus. General anesthesia was administered with tracheal intubation during the surgical procedure. The patient was positioned in the lithotomy position, underwent standard disinfection and draping, and a catheter was inserted into the affected side ureter. Subsequently, a ureteral catheter was left in position after reaching the renal pelvis. The patient is positioned in the prone position with the waist elevated in the renal area. Under the guidance of B ultrasound, the appropriate puncture point was selected between the 11 or 12 intercostal margins, the scapular line and the posterior axillary line. After successful puncture, the zebra guidewire (COOK, USA) (0. 035 inches) was introduced, and the fascia expander was used to distill the percutaneous nephrolithotomy channel. After the percutaneous nephroscope entered the kidney, the holmium laser system (PowerSuite) was used to launch the holmium laser to smash the stones, the large stones were removed with stone forceps, and the small stones were flushed out with sodium chloride injection. Following the operation, a double J tube and a nephrostomy tube were routinely inserted. On the third day after the operation, an abdominal CT scan was performed for reexamination. The nephrostomy tube was removed on the fifth day after the operation, while the double J tube was removed one month after the operation, and the perioperative nursing was provided accordingly during the period.

Group B was treated with PCNL under the guidance of holographic image technology. (1) Select equipment: Holographic image 3D reconstruction model/endoscopic image comprehensive analysis software system (Beijing Renxin Medical Technology Co., LTD., Beijing, China), rigid ureteroscope (Wolf, Germany), nephroscope (Wolf, Germany), flexible ureteroscope (STORZ, Germany), guide wire (COOK, America), holmium laser system (America) Power- Suite), 3D tracking glasses (zSpace, USA), mixed reality glasses (Holoens, South Logan, UT, USA).

(Millipore, Billerica, MA, USA) (Roche, Basel, Switzerland)), etc. (2) Holographic image construction. (3) Preoperative planning. Preoperative planning of the puncture channel and puncture route, the specific steps are as follows: 1) According to the results of holographic images, the situation of stones, renal pelvis and renal calices were analyzed, and the target renal calices that could be removed with the greatest effect were selected. 2) The renal papilla of the target calices was identified and located, revealing clear visualization of the renal blood vessels combined with display of the holographic image. Caution must be exercised to prevent puncturing the blood vessels along the puncture route, ensuring that the needle is inserted from the renal papilla. 3) The positioning of the skin puncture point is determined using body surface landmarks. This point is then connected in a straight line to the target renal calyx, forming the route for the puncture channel. 4) Index measurement. The indicators to be measured include the length of skin puncture point to renal calyceal, puncture route and skin Angle, distance between target calyceal and upper and lower edge of kidney, etc. (4) Preoperative simulation of puncture. Once the puncture channel and route had been established, clinicians were able to utilize 3D tracking glasses and employ the holographic image-based model to simulate the puncture procedure on the zSpace machine. The simulation allowed for the evaluation of the targeted puncture calyx, as well as the calculation of the optimal puncture depth and angle. (5) Intraoperative assistance. With clear identification of the skin puncture point and puncture angle, the ultrasound probe can be effectively utilized for swift intraoperative localization. In cases where artificial hydronephrosis was present with a ureteral catheter in place, the skin puncture point remained unchanged, however, slight adjustments in the needle-to-skin angle can be made in conjunction with the assistance of the ultrasound probe. Following a successful puncture, the procedure was carried out in accordance with the routine PCNL protocols of Group A, with the inclusion of a prepared flexible ureteroscope during the operation. In the case of encountering a calyceal stone that is parallel to the puncture channel, a flexible ureteroscopy with holmium laser lithotripsy was utilized via the percutaneous renal access. Following the procedure, the nephrostomy tube and double J tube were inserted, and an abdominal CT scan was reexamined prior to the removal of the double J tube one month after the operation.

### Observation indicators

**Figure 1:**
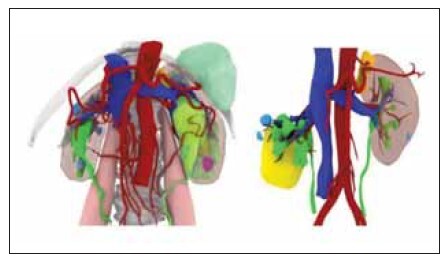
Hologram technology can specifically observe renal blood vessels, renal pelvis and renal lamps.

**Figure 2:**
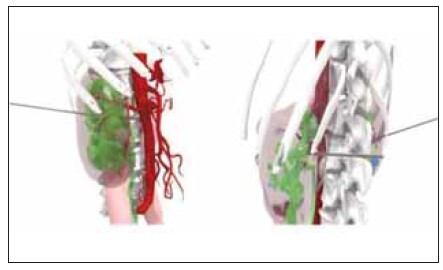
Preoperative evaluation of stones, renal pelvis, and renal calyx and selection of puncture target lamps.

**Figure 3:**
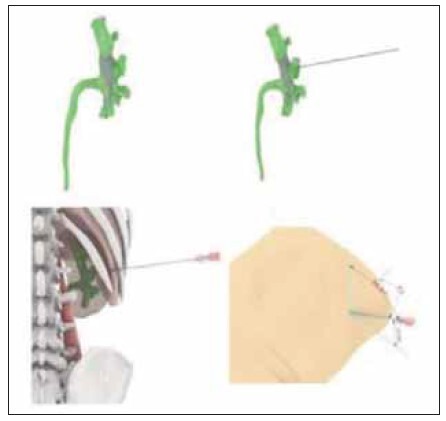
Location of the skin puncture point.

(1) Relevant surgical indicators. Record surgical indicators, including puncture time for target caly ceal, operation time, and postoperative hemoglobin decline. (2) Stress response indicators. We obtained venous blood samples of 3–5 mL before and 24 h post-surgery, following an overnight fast starting from 22:00 the night before. The serum supernatant samples were then subjected to centrifugation using a 3K- 10 multifunctional centrifuge (provided by Sigma Corporation, St. Louis, MO, USA) at 3000 r/min speed, 5 cm centrifugal radius, and 15 minutes centrifugation time. Using chemical colorimetry, we measured the levels of superoxide dismutase (SOD), malondialdehyde (MDA), and glutathione peroxidase (GSH-Px) in the serum (kit: Nanjing Xindi Biological Pharmaceutical Engineering Co., LTD., Nanjing, China). (3) A serum supernatant sample was obtained through centrifugation of 3–5 mL of fasting venous blood collected from both groups prior to and 24 h following surgery. Tumor necrosis factor-1 (TNF-1), interleukin-6 (IL-6), and IL-1 levels were quantified using an enzyme-linked immunosorbent assay (ELISA) (kit: Shanghai Enzyme Linked Biotechnology Co., LTD., Shanghai, China). Additionally, the levels of tumor necrosis factor-α (TNF-α), interleukin-6 (IL-6), and IL-1 were determined through immunoturbidimetry (kit: Shanghai Jingkang Biological Enginee ring Co., LTD., Shanghai, China) to detect high-sensitivity Creactive protein (hs-CRP) levels. (4)A automatic biochemical analyzer (model: BK-200, purchased from Jinan Oulaiboko Instrument Co., Ltd., Jinan, China) was used to detect the patient’s serum neutrophil gelatinase-associated lipocalin (NGAL) before surgery and 24 h after surgery. Double-antibody sandwich enzyme-linked immunosorbent assay (ABC-ELISA) was used to detect serum induced protein 8-like molecule 2 (TIPE2) levels, and immunoturbidimetric method was used to detect β2-microglobulin (β2-MG) levels. (5) Renal function indicators. The levels of serum creatinine (SCr), blood urea nitrogen (BUN), cystatin C (CysC) were measured by automatic biochemical analyzer before operation and 24 h after operation. (6) Incidence of complications. The occurrence of adjacent organ injury, severe bleeding, septic shock, and renal failure within 1 month after operation was recorded. (7) One-time stone clearance rate. The stone clearance was reexamined at 1 month after operation. Figure 1 Figure 2 Figure 3

### Statistical analysis

Statistic Package for Social Science (SPSS) 22.0 software (IBM, Armonk, NY, USA) was used to process the data. Measurement data (related surgical indicators, oxidative stress, response indicators, inflammatory response indicators, renal injury factors, renal function indicators) were expressed as (x̄±S), and t test was used. Count data were expressed as n (%) (complication rate, stone clearance rate), using 2 test, P < 0.05 was considered statistically significant.

## Results

### Surgery-related indicators

**Figure 4:**
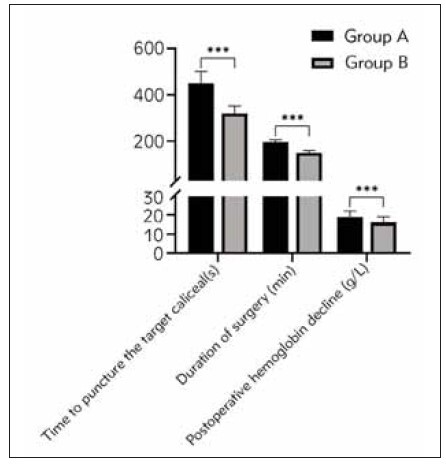
Two sets of surgical related indicators <br>(***P<0.001).

Group B exhibited shorter puncture times for the target caliceals and operation durations compared to group A. Furthermore, a significant decrease in intraoperative blood loss was observed in group B in comparison to group A (*P*<0.05). See Table 2, Figure 4.

**Table 2:** Two groups of surgery-related indicators (x̄±S).

Group	n	Time to <br>puncture the <br>target caliceal <br>(s)	Duration of <br>surgery (min)	Postoperative <br>hemoglobin <br>decline (g/L)
group A	40	450.86±50.25	195.35±10.84	18.70±3.20
group B	30	320.49±30.99	150.84±8.52	16.11±2.71
P		0.000	0.000	0.000

### Stress response indicators and sCr, BUN and CysC

**Figure 5:**
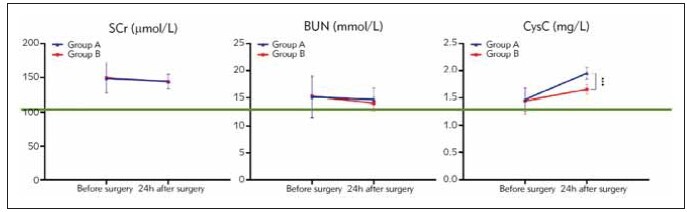
Two sets of oxidative stress response indicators (*P<0.05, **P<0.01).

There were no statistically significant differences in serum levels of SOD, MDA, and GSH-Px between group A and group B before the operation (*P*>0.05). However, 24 h after surgery, group B demonstrated higher levels of SOD and GSH-Px compared to group A, while exhibiting lower levels of MDA (*P* < 0.05). See Table 3, Figure 5.

**Table 3:** Oxidative stress, response indexes of the two groups before and after operation (x̄±S).

Group	n	SOD (U/mL)		MDA (nmol/mL)		GSH-Px (U/L)	
		Before surgery	24h after surgery	Before surgery	24h after surgery	Before surgery	24h after surgery
group A	40	700.43±100.85	600.38±110.26^a^	6.38±0.59	8.83±0.62^a^	90.41±10.56	72.59±10.24^a^
group B	30	710.27±110.96	670.53±110.20^a^	6.42±0.48	8.39±0.76^a^	90.28±10.93	81.08±12.93^a^
P		0.700	0.010	0.763	0.010	0.960	0.003

### Inflammatory response indicators

**Figure 6:**
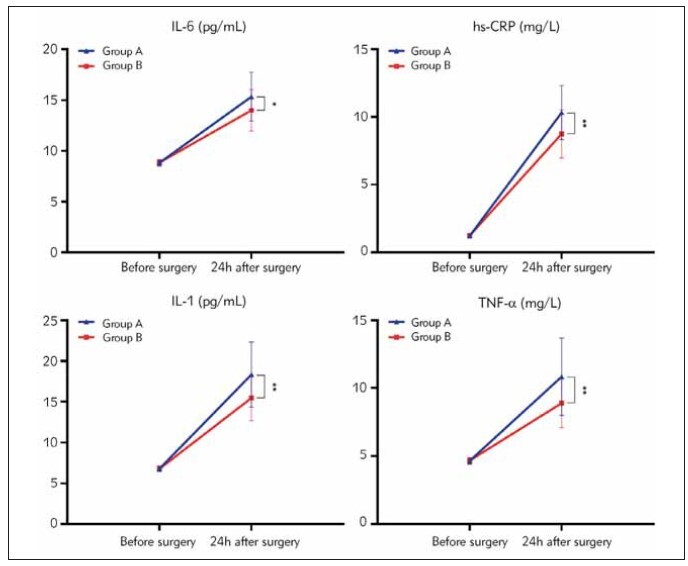
Two sets of inflammatory response indicators (*P<0.05,**P<0.01).

There were no statistically significant differences in preoperative inflammatory response indices between group A and group B (*P* > 0.05). However, 24 h after surgery, group B exhibited lower levels of TNF-, IL-6, IL-1, and hs-CRP compared to group A (*P* < 0.05). See Table 4, Figure 6.

**Table 4:** Two sets of inflammatory response indicators before and after surgery (x̄±S).

Group	n	IL-6 (pg/mL)		hs-CRP (mg/L)		IL-1 (pg/mL)		TNF-α (mg/L)	
		Before <br>surgery	24 h after <br>surgery	Before <br>surgery	24 h after <br>surgery	Before <br>surgery	24 h after <br>surgery	Before <br>surgery	24 h after <br>surgery
group A	40	8.82±0.32	15.32±2.42^a^	1.22±0.15	10.32±1.99^a^	6.78±0.35	18.32±3.99^a^	4.65±0.25	10.84±2.85^a^
group B	30	8.84±0.30	13.99±2.05^a^	1.24±0.12	8.75±1.78^a^	6.77±0.33	15.45±2.78^a^	4.68±0.23	8.89±1.79^a^
P		0.791	0.018	0.551	0.001	0.904	0.001	0.609	0.002

### Renal injury factors levels

**Figure 7:**
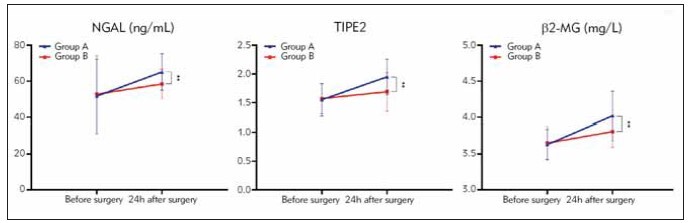
Renal injury factor levels in two groups of patients (**P<0.01).

Prior to the operation, there were no statistically significant differences in NGAL, TIPE2, and β2- MG levels between group A and group B (*P*>0.05). However, 24 h after the operation, group B demonstrated lower levels of NGAL, TIPE2, and β2-MG compared to group A (*P* < 0.05). See Table 5, Figure 7.

**Table 5:** Renal injury factor levels before and after surgery in two groups of patients (x̄±S).

Group	n	NGAL (ng/mL)		TIPE2		β2-MG (mg/L)	
		Before <br>surgery	24h after <br>surgery	Before <br>surgery	24h after <br>surgery	Before <br>surgery	24h after <br>surgery
group A	40	51.61±20.55	65.05±10.25^a^	1.55±0.28	1.95±0.31^a^	3.63±0.21	4.02±0.34^a^
group B	30	52.68±21.50	58.37±8.18^a^	1.57±0.26	1.69±0.33^a^	3.65±0.23	3.81±0.22^a^
P		0.838	0.005	0.761	0.001	0.706	0.004

### Renal function indexes

**Figure 8:**
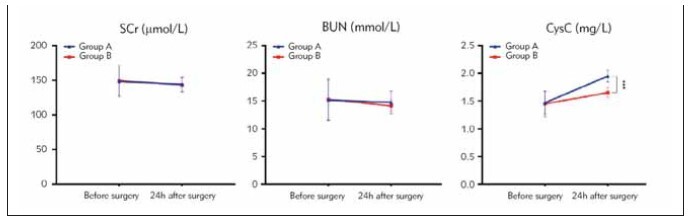
Renal function indicators in two groups of patients (***P<0.001).

There was no statistically significant difference observed in the levels of SCr, BUN, CysC between group A and group B before the operation (*P* > 0.05). 24 h after the operation, There was no statistically significant difference observed in the levels of SCr, BUN, between group A and group B (*P* > 0.05); however, exhibited lower levels of CysC compared to group A (*P* < 0.05). See Table 6, Figure 8.

**Table 6:** Renal function indicators before and after surgery in two groups of patients (x̄±S).

Group	n	SCr (μmol/L)		BUN (mmol/L)		CysC (mg/L)	
		Before surgery	24 h after <br>surgery	Before surgery	24 h after <br>surgery	Before surgery	24 h after <br>surgery
group A	40	148.22±20.54	159.62±22.71^a^	15.16±3.67	16.79±3.58^a^	1.47±0.21	1.95±0.11^a^
group B	30	149.24±22.52	161.19±23.26^a^	15.31±3.74	17.22±3.71^a^	1.45±0.23	1.65±0.09^a^
P		0.844	0.778	0.867	0.626	0.706	0.000

### Incidence of complications

There was no statistically significant difference observed in the incidence of postoperative complications between group B and group A (*P* > 0.05). See Table 7.

**Table 7:** Two groups of postoperative complications n (%).

Group	n	The adjacent organs <br>were injured	Severe bleeding	Septic shock	Renal Failure	Total
group B	40	1 (2.50)	3 (7.50)	1 (2.50)	0 (0.00)	5 (8.00)
group B	30	0 (0.00)	0 (0.00)	0 (0.00)	0 (0.00)	0 (0.00)
χ^2^						2.374
P						0.123

### One-time stone clearance rate

One month after operation, the rate of one-time stone clearance in group B was 93.33% (28/30), which was higher than the rate in group A of 75.00% (30/40) ( 2=4.057, *P* < 0.05).

## Discussion

Due to the large total stone burden, irregular location and shape of complex kidney stones, the treatment is difficult and the medical cost is high. At present, PCNL is the mainstream treatment method [7] [8]. Some studies have pointed out that complex renal calculi are often accompanied by abnormalities in the structure of the renal collecting system and the course of renal blood vessels, which vary greatly from patient to patient, and the puncture point and puncture channel set by PCNL are different for each patient, which should be different from person to person [9] [10]. Other studies have pointed out that complex kidney stones are often accompanied by abnormal renal anatomy, especially in horseshoe kidney patients, due to renal fusion, malrotation and abnormal vascular supply, conventional ultrasound and fluoroscopy are difficult to locate [11]. Therefore, the preliminary evaluation of routine renal CT examination before PCNL for patients with complex renal calculi combined with intraoperative ultrasound and fluoroscopy cannot meet the actual needs of patients, and the clinical application has certain limitations. Therefore, it is still necessary to seek more efficient guidance methods to carry out individualized PCNL treatment according to the specific conditions of patients.

The rapid advancement of artificial intelligence and digital medicine has led to the emergence of promising applications of augmented reality and virtual reality in urology [12] [13]. According to this study, group B demonstrated shorter puncture and operation times compared to group A, along with reduced intraoperative blood loss. These findings highlight the potential of holographic image-guided PCNL to decrease blood loss, puncture time, and operation time associated with the management of complex renal calculi. The reasons are as follows: (1) Holographic imaging technology is based on the DICOM data of preoperative enhanced CT of patients, and holographic reconstruction is carried out after visual detection, segmentation, extraction, measurement, verification and calculation. The reconstructed model is translucent, which can intuitively and three-dimensionally display the relationship between stones, collecting systems, and renal blood vessels, thereby effectively distinguishing normal bones, blood vessels, muscles and other tissues in the target area. The surgeon can accurately understand the distribution of renal arteries and veins, the relationship between the collecting system and the stone, and then design the puncture channel reasonably through the holographic image results. In addition, simulation puncture can be performed before the operation, which reduces the risk of calyceal neck tear when the nephroscope swings extensively during the operation, and is conducive to shortening the puncture time of the target calyceal [14]. (2) In addition, through holographic image reconstruction, the surgeon can fully understand a specific anatomical structure space by observing the relationship between renal vessels, renal calyceal and stones, which is convenient for the surgeon to predict the intraoperative situation before the operation, and prepare for the corresponding operation, which is conducive to the smooth implementation of the operation and shorten the operation time [15] [16]. (3) Simultaneously, preoperative holographic image technology enables the rational design of an optimal puncture route. Under the guidance of holographic image technology, the operator can minimize unnecessary harm to the renal collecting system, surrounding organs, and blood vessels by utilizing intraoperative holographic image navigation and positioning technology, in addition, the risk of intraoperative blood loss can be effectively reduced [17] [18].

Some studies have pointed out that traumatic operation can induce oxidative stress in the body, and severe oxidative stress can activate sympathetic nerve and renin-angiotensin-aldosterone system, leading to imbalance of internal environment homeostasis, and then affect the endocrine system, nervous system and immune system [19] [20] [21]. The reason for this is that holographic images obtained by holographic image reconstruction can be rotated and scaled at full angles, and specific organs, tissues or blood vessels can be arbitrarily hidden, split or displayed according to needs [22]. The surgeon can fully understand the size and location of the stone, the adjacent relationship of the important tissues and organs around the stone, and the specific blood vessels around the stone before operation, which is helpful to choose a more appropriate puncture point and puncture channel. Furthermore, with the assistance of intraoperative holographic image navigation and positioning technology, precise targeting of the holmium laser for stone fragmentation can be achieved, alongside the facilitation of meticulous execution of other surgical procedures. This approach proves beneficial in mitigating surgical trauma, and holds significant implications for reducing postoperative stress and inflammatory responses [23].

Several studies have emphasized the need for precise and minimally invasive techniques in urology surgery, particularly when performing renal hilar surgery. Inadequate surgical procedures can lead to postoperative renal dysfunction, resulting in compromised surgical outcomes and decreased the one-time stone clearance rate [24]. The study findings revealed that, 24 h after operation, group B exhibited lower levels of NGAL, TIPE2, and β2-MG compared to group A. Similar trends were observed for CysC, with group B showing lower levels compared to group A at the same time point. Additionally, during reexamination one month after the operation, group B demonstrated a higher rate of one-time stone clearance compared to group A. These findings provide confirmation that utilizing holographic image technology in the management of complicated renal calculi via PCNL can effectively decrease the presence of renal injury markers, enhance renal function, and augment the rate of one-time stone clearance. The rationale behind these outcomes can be attributed to the following factors. (1) Under the guidance of holographic image technology, PCNL has less intraoperative blood loss, shorter operation time, less adverse effects on renal blood and oxygen supply function, which is conducive to reducing kidney injury and better recovery of renal function after operation. (2) PCNL requires systematic preoperative design discussion, accurate intraoperative analysis and positioning, and perfect postoperative evaluation, which is difficult for doctors to master in a short time. Guided by holographic imaging technology, it facilitates a comprehensive understanding of the distribution of renal arteries and veins. Moreover, it elucidates the relationship between the collection system and stones, as well as clarifies the interplay between renal blood vessels, renal calyces, and stones. This aids in formulating appropriate surgical plans and enhancing physicians’ comprehension of the disease, thus enhancing the safety and effectiveness of the operation, reducing kidney injury, and promoting early recovery of renal function.

Relevant studies have pointed out that PCNL under the guidance of holographic image technology can effectively reduce the incidence of related complications, avoid secondary or even multiple stone clearing operations in the later stage, effectively save medical insurance funds and reduce family economic burden [25]. In terms of complications in this study, all patients in group B successfully underwent the operation without any serious complications, such as postoperative bleeding, injury to adjacent organs, or septic shock. On the other hand, in group A, there were 1 case of adjacent organ injury, 3 case of severe bleeding, and 1 case of septic shock. Although the incidence of complications in group B was lower than that in group A, the difference was not statistically significant. These findings differ from the conclusions drawn in the aforementioned study. Upon analysis, this difference may be attributed to the limited number of cases, shorter follow-up duration, and the implementation of scientifically designed perioperative nursing care in both groups, which proved to be effective in minimizing complications. In theory, according to the positioning of body surface landmarks such as the twelve costal, the holographic model and the real surgical area are superimposed in equal proportion. The surgeon wearing mixed reality glasses can quickly locate and establish the puncture channel according to the preoperative planning and design. However, in the early studies of our center, it was found that it was difficult to completely match and integrate the holograms with the real surgical area due to the interference of breathing and body position changes, in addition, there were problems such as dizziness after wearing mixed reality glasses, and it was difficult to quickly and accurately establish percutaneous renal access, all of which would have adverse effects on the research results. Therefore, in the future, our center will cooperate with a number of hospitals in the regional medical alliance to increase the sample size and lengthen the follow-up time, and conduct a multi-center randomized controlled study to conduct an in-depth analysis of the safety and effectiveness of PCNL surgery in the treatment of complex kidney stones under the guidance of holographic imaging technology, in order to obtain more scientific, accurate and comprehensive research results.

In conclusion, the utilization of holographic image technology in guiding PCNL for treating complex renal calculi offers several advantages. It can shorten the puncture time of the target calyx and operation duration, minimize intraoperative blood loss, effectively reduce postoperative stress and inflammatory responses, and decrease the levels of kidney injury factors. These benefits contribute to the enhancement of renal function and an increase in the rate of successful stone clearance. Overall, the clinical application of this approach has demonstrated positive outcomes.

## Dodatak

### Ethical Compliance

This study was approved by the ethics committee of Mindong Hospital Affiliated to Fujian Medical University (Approval no. 2022081701k). Signed written informed consents were obtained from the patients and/or guardians.

### Funding

This work was supported by the Project supported by the Medical Innovation Foundation of the Fujian Provincial Health Commission, China (Grant No.2021CXA048); Project supported by the Natural Science Foundation of Fujian Provincial, China (Grant No.2023J011907 ).

### Conflict of interest statement

All the authors declare that they have no conflict of interest in this work.
